# Correcting nucleotide-specific biases in high-throughput sequencing data

**DOI:** 10.1186/s12859-017-1766-x

**Published:** 2017-08-01

**Authors:** Jeremy R. Wang, Bryan Quach, Terrence S. Furey

**Affiliations:** 1 Department of Genetics, University of North Carolina at Chapel Hill, CB 7032, 7314 Medical Biomolecular Research Building, 111 Mason Farm Road, Chapel Hill, 27599 NC USA; 2 Department of Genetics, University of North Carolina at Chapel Hill, Chapel Hill, NC USA; 3 Department of Biology, Carolina Center for Genome Sciences, Lineberger Comprehensive Cancer Center, University of North Carolina at Chapel Hill, Chapel Hill, NC USA

**Keywords:** Epigenomics, Bias correction, DNase-seq, ATAC-seq, ChIP-seq, FAIRE-seq

## Abstract

**Background:**

High-throughput sequence (HTS) data exhibit position-specific nucleotide biases that obscure the intended signal and reduce the effectiveness of these data for downstream analyses. These biases are particularly evident in HTS assays for identifying regulatory regions in DNA (DNase-seq, ChIP-seq, FAIRE-seq, ATAC-seq). Biases may result from many experiment-specific factors, including selectivity of DNA restriction enzymes and fragmentation method, as well as sequencing technology-specific factors, such as choice of adapters/primers and sample amplification methods.

**Results:**

We present a novel method to detect and correct position-specific nucleotide biases in HTS short read data. Our method calculates read-specific weights based on aligned reads to correct the over- or underrepresentation of position-specific nucleotide subsequences, both within and adjacent to the aligned read, relative to a baseline calculated in assay-specific enriched regions. Using HTS data from a variety of ChIP-seq, DNase-seq, FAIRE-seq, and ATAC-seq experiments, we show that our weight-adjusted reads reduce the position-specific nucleotide imbalance across reads and improve the utility of these data for downstream analyses, including identification and characterization of open chromatin peaks and transcription-factor binding sites.

**Conclusions:**

A general-purpose method to characterize and correct position-specific nucleotide sequence biases fills the need to recognize and deal with, in a systematic manner, binding-site preference for the growing number of HTS-based epigenetic assays. As the breadth and impact of these biases are better understood, the availability of a standard toolkit to correct them will be important.

**Electronic supplementary material:**

The online version of this article (doi:10.1186/s12859-017-1766-x) contains supplementary material, which is available to authorized users.

## Background

High-throughput short-read sequencing (HTS) has enabled the genome-wide identification of functional regulatory regions including transcription factor binding sites and epigenomic features such as histone tail modifications and regions of open chromatin. HTS-based assays such as ChIP-seq, DNase-seq, FAIRE-seq, and ATAC-seq generate millions of reads per experiment that then are used to identify regions of interest. However, a combination of biases in these HTS protocols often results in a deviation from the background frequency of nucleotides present at each position in HTS reads, which we call nucleotide-specific bias. As the routine use of HTS is already widespread and increasing, it is especially important to fully understand any biases associated with HTS protocols and take these biases into account when analyzing the resulting data [1].

There are several steps involved in preparing pools of DNA for HTS, each of which may introduce nucleotide-specific bias. All short-read HTS protocols require some form of DNA fragmentation into smaller DNA molecules to facilitate high-throughput sequencing. In many of these assays, including ChIP-seq and FAIRE-seq, this is accomplished by sonication. There is evidence that sonication breaks DNA strands between nucleotides preferentially based on their binding affinity [2]. Most assays also use adapter-mediated polymerase chain reaction (PCR) to amplify DNA before sequencing. The adapters used in this step must be ligated to the ends of DNA fragments to enable PCR amplification. Although these adapters are ligated to blunt-end DNA, slight nucleotide-specific ligation preferences may create noticeable biases in the amplified DNA and resulting sequence data [3].

In addition, there are a variety of assay-specific steps that may introduce nucleotide biases. In DNase-seq [4], the DNase I restriction enzyme preferentially digests DNA in nucleosome-depleted regions of chromatin. Ideally, DNase I cleaves DNA randomly within this open chromatin, but it has been shown [5, 6] that DNase I exhibits significant nucleotide-specific cleavage biases. Likewise, other selective assays including chromatin immunoprecipitation (ChIP), formaldehyde-assisted isolation of regulatory elements (FAIRE) [7], and assay for transposase-accessible chromatin (ATAC) [8] include assay-specific steps that may introduce nucleotide-specific biases. It is difficult to pinpoint exactly which of these contribute to nucleotide-specific biases within a given assay since the read sequence is available only upon completion of all steps. Therefore, it is preferable to identify the pattern of nucleotide-specific bias without attributing it to a particular source and assign weights to reads that implicitly correct for all observed biases.

Much of the previous work on correcting biases in HTS data has focused on RNA-seq [3, 9–11]. Sequencing biases in RNA-seq data prevent the accurate estimation of relative transcript abundances. These methods focus on correcting relative transcript abundances as a whole, based on the effect of bias within exons. As such, these methods are unsuitable for adjusting biases on a read-by-read basis and do not perform as well in a genomic DNA context as opposed to RNA.

Recently, methods have been proposed for correcting nucleotide-specific biases in DNase-seq data. The accurate estimation of cut frequencies in DNase-seq is particularly important in the identification of “footprints”, which correspond to evidence of transcription-factor binding characterized by local dips in digestion within larger DNase peaks [12]. These methods focus on correcting only bias introduced by the nucleotide-specific preferences in DNase I binding and cutting. [13] use deproteinized “naked” DNA to identify a signature of cleavage bias independent of chromatin structure. This approach requires extensive sequencing to estimate these well and is highly sensitive to experimental conditions and lab or batch effects under which both the regular DNase-seq and “naked” DNase-seq is performed. Additionally, this and other methods [6, 14] only characterize DNase-seq bias within a small window (2-6 bp) surrounding the DNase I binding site and fail to account for biases at other locations in the read and biases due to other factors. It should be noted that existing bias correction methods and the method we propose do not correct sequencing errors in reads, and “correct” for biases by reweighting reads or loci, not by changing nucleotides in the read.

Similarly, methods have been published to address sequence bias in ChIP-seq data by taking into account the contribution of GC content, chromatin structure, and other factors [15]. However, this approach accounts only for a specific subset of biases and requires a prohibitive collection of DNase-seq data, mappability and GC measures, and two ChIP-seq controls.

We introduce a method that corrects nucleotide-specific bias in HTS from a variety of DNA-based sequencing assays. Our method computes an accurate baseline nucleotide distribution within the same sample data without the need for extra sequencing and corrects biases that are based on nucleotide composition within and surrounding HTS reads, regardless of the source of bias. We calculate read weights that adjust the distribution of position-specific nucleotide frequencies within the read to match the expected nucleotide frequency based on a random sampling of reads within the target region(s). We demonstrate that this adjustment improves the performance of each of the evaluated protocols for detecting genomic features, including open chromatin regions and transcription-factor binding footprints.

## Methods

**Fig. 1:**
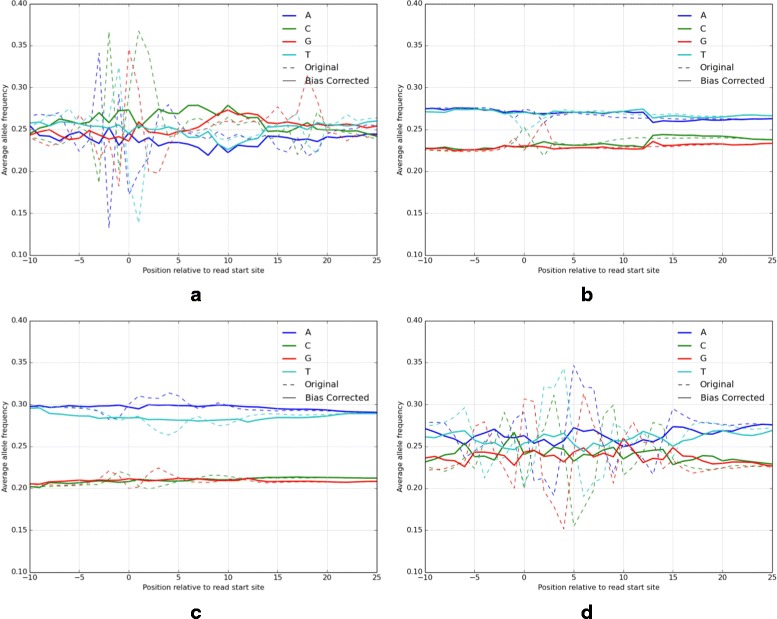
Read-relative position-specific nucleotide frequencies before and after bias correction. *Dotted lines* show significant position-specific nucleotide bias, most evident immediately surrounding the read start site (0). The *solid lines* show the nucleotide frequencies after bias correction. **a** DNase-seq, **b** ChIP-seq, **c** FAIRE-seq, **d** ATAC-seq

Sequence reads from a variety of HTS assays, including DNase-seq, ChIP-seq, FAIRE-seq, and ATAC-seq show distinct position-specific nucleotide biases that differ across assays (Fig. 1). The observable nucleotide bias may result from a number of inseparable sources of bias specific to a particular assay or to a HTS protocol, including sonication, digestion by selective restriction enzymes, and adapter-mediated PCR. The final read sequence from these experiments reflects a summation of these factors that cannot be easily disentangled, if at all. Some of these biases are shared across assays, for instance from the use of a common fragmentation technique or HTS technology. The degree, position, and nucleotide distribution of biases vary widely across assay-type (Fig. 1).

**Fig. 2:**
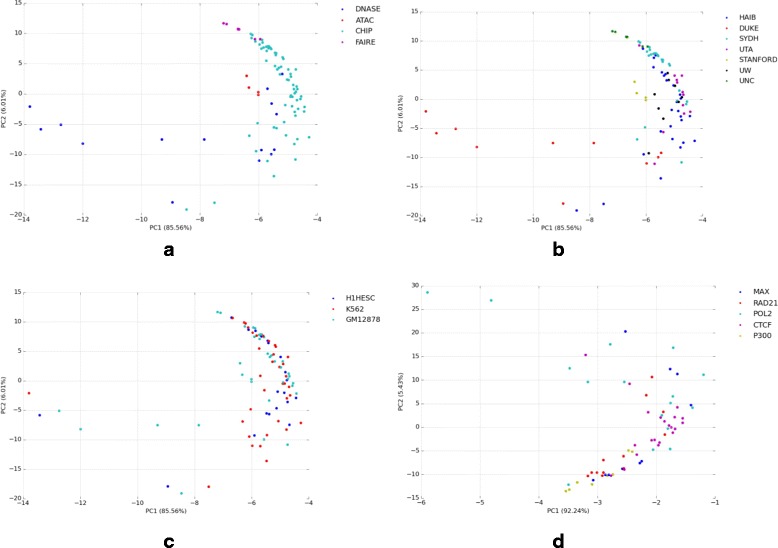
Principal component (PC) analysis of 5-mer frequencies shows clear distinctions between DNase-seq, ChIP-seq, FAIRE-seq, and ATAC-seq (**a**). Secondarily, clustering is evident by the lab which ran the experiment (**b**) (ENCODE production groups, see http://genome.uwencode.org/ENCODE/contributors.html; HAIB: HudsonAlpha, DUKE: Duke University, SYDH: Stanford/Yale/UCDavis/Harvard, UTA: University of Texas Austin, STANFORD: Stanford University, UW: University of Washington Seattle, UNC: University of North Carolina Chapel Hill). No clustering is observed by cell type (**c**) or by transcription factor (in ChIP-seq experiments) (**d**)

To characterize the biases within and differences between experiments, we computed the frequency of every *k*-mer in each non-overlapping *k* nucleotide window as described in “Computing nucleotide bias” section. We used the full set of (*f*_*k-mer*_,*r+ik*), where *f*_*k-mer*_ is the relative frequency of a *k*-mer at an offset *i*∗*k* from the aligned read location *r*, as our feature space to perform principal component analysis (PCA). Figure 2 shows the PCA across several ENCODE experiments. The first two components, describing more than 92% of the variation, show clustering by assay type (Fig. 2 a) and the lab/investigators (Fig. 2 b) who performed the experiments, indicating that we are seeing true biases based on the experimental protocol used. Additionally, we do not observe any noticeable clustering by cell type (Fig. 2 c) or transcription factor (Fig. 2 d) (among ChIP-seq experiments), which would both be evidence that we are mistaking true biological signal for bias. We characterize and correct biases within each read, and also consider nucleotides upstream and downstream of the read in the reference genome to take into account the larger sequence context. This is necessary due to biases seen in sonication, DNase I digestion, and other steps that break DNA, which are dependent on the full sequence surrounding the break site.

We observed the greatest cumulative bias in DNase-seq and ATAC-seq (Fig. 1). The bias we observed across DNase-seq experiments mirrored that described previously [6, 13, 14]. Most notably, we saw the greatest nucleotide variance across a hexamer at the 5’ end of the read, indicative of DNase-I binding preference (see Fig. 1 a. ATAC-seq also has a large, recently characterized [16], assay-specific bias. Figure 1 d illustrates a symmetrical nucleotide bias centered between nucleotides 4 and 5. The Tn5 transposon used in ATAC-seq was previously observed [17] to selectively integrate at a 9bp short direct repeat (SDR). We observe this symmetrical Tn5 binding preference in the aggregate ATAC-seq read profile. The most over-represented motif we found to be GGTTT/AAACC, consistent with the SDR predicted by [17], GTTT(T/A)AAAC (see Fig. 1 d.

Our bias correction method is applied independently for each replicate or sequencing run, since each may have its own unique biases. Briefly, we compute the frequency of *k*-mers (motifs of length *k*) starting at each position relative to the start of the aligned reads, including genomic positions upstream and downstream of the reads. For brevity, we call the sliding *k*-mer windows at each relative position “tiles”, where every aligned read has a specific *k*-mer at the same “tile” relative to their respective aligned start position. Next, we compute expected baseline *k*-mer frequencies by sampling randomly from within all reads and a 50-bp margin around each read. This baseline exhibits no significant position-specific nucleotide variance while capturing the expected average nucleotide content of the sequenced feature(s), such as average GC content, in genomic regions being targeted in a particular assay. From this set of tiles, we identify those that are significantly biased - where variance is above the 95% confidence threshold of the baseline variance. The pairwise covariance between *k*-mer frequencies of all biased tiles is computed. The frequencies of correlated tiles are averaged together; then all independently varying tile groups are compounded to produce an overall read weight. To adjust these weights to reflect the local likelihood of observing a read at a particular locus, we normalize the overall weight by the average weight of simulated reads at every locus within a 20 bp window surrounding the observed read site.

Our method is open source and freely available at http://github.com/txje/sequence-bias-adjustment.

### Samples and data

We ran and evaluated our method using whole-genome DNase-seq, ChIP-seq, FAIRE-seq, and ATAC-seq. To observe effects of biases in sample, preparation, and protocol, we used data from GM12878, K562, and H1-hESC cell lines and from several different labs and institutions. Sequence data from several open chromatin and transcription factor binding assays were selected from the Encyclopedia of DNA Elements (ENCODE) project [18], including DNase-seq, ChIP-seq, and FAIRE-seq from GM12878, H1-hESC, and K562. ATAC-seq data from GM12878 (GSE47753) [8] was also downloaded from GEO. To assess the effect of bias correction on uniformly digested whole-genome DNA, we used DNase-seq data from deproteinized “naked” K562 DNA (GSM1496625). All of these data were previously aligned to the GRCh37/hg19 human reference genome.

### Computing nucleotide bias

We first detect the extent of nucleotide-specific biases within and surrounding all aligned reads, *R*. Nucleotide-specific bias is quantified by the variance in relative frequency of each nucleotide at a particular locus relative to the 5’ end of a read, *r*. We confirmed that nucleotide bias observed in aligned sequences was not a result of bias in the alignment protocol by comparing intra-read nucleotide content for all reads with the nucleotide content on the reference genome where reads align. These showed identical patterns of bias, indicating that no strong nucleotide bias is introduced during alignment. Throughout, we used the nucleotide sequence of the reference genome, *S*, to take into account bias outside the read boundaries.

We calculate the bias signature by computing the frequency ($f_{kmer}^{t}$) of *k*-mers across each read. For each offset from −20 to *n*+20 relative to the read’s alignment start position, *A*(*r*), in *S*, where *n* is the read length, we count the occurrences of each unique *k*-mer across all reads. Each count is then divided by the total number of reads to give the relative frequency of that *k*-mer; these frequencies represent the global bias signature for a single experiment (Eq. 1). We chose a value of *k* to balance the number of reads/power and correction accuracy. Throughout this paper, we used *k*=5, although values from 4-6 were evaluated and made little difference. If the method were applied to data with very low coverage, a lower value of *k* could be chosen to improve the power to estimate each *k*-mer frequency. Likewise, a larger value of *k* could be used to improve the correction accuracy if sufficient data exists to compute confident *k*-mer frequency estimates. To increase *k* by 1, four times as many reads are required to reach the same sampling power.

1 $$ f_{{kmer}}^{t} = \frac { \sum\limits_{r\:\in\:R} \left\{ \begin{array}{ll} 1, & \text{if } {S_{\left[A(r)+t, A(r)+t+|kmer|\right]} = {kmer}} \\ 0, & \text{otherwise} \end{array}\right.} {|R|}  $$

### Computing baseline nucleotide frequencies

Baseline *k*-mer frequencies are sampled randomly from the reference sequence relative to the density of aligned reads. For each observed read, a number of “pseudo-reads” are sampled randomly in the region of −25 to *n*+25 relative to the read start position, where *n* is the read length. The sampling is uniform within the given window, but the number of samples taken is equal to the total number of aligned reads in the window. This has the effect of sampling the baseline exponentially relative to the read density, amplifying the contribution of higher coverage regions and helping to reduce the effect of isolated and erroneous reads. Each of the baseline sampled “pseudo-reads” is used to accumulate *k*-mer frequencies as described in the previous section and in Eq. 2, where *x* is a random variable from *X*∼*U*(−25,*n*+25).

2 $${} {{\begin{aligned} b_{{kmer}} \,=\, \frac {\sum\limits_{r\:\in\:R} {\frac{\sum\limits_{i\in \left[0,|r'\in R, A(r)-25 \le A(r') < A(r)+n+25|\right]} \left\{\begin{array}{ll} 1, & \text{if } {S_{\left[A(r)+x, A(r)+x+|kmer|\right]} = {kmer}} \\ 0, & \text{otherwise} \end{array}\right.} {|r'\in R, A(r)-25 \le A(r') < A(r)+n+25|}}} {|R|} \end{aligned}}}  $$

### Computing read weights

To compute read weights from bias and baseline *k*-mer frequencies is nontrivial, largely because bias is not uniform across reads and bias values are not independent between *k*-mer windows, or “tiles”. We often observe high covariance between correction weights for both adjacent (abutting but non-overlapping) and non-adjacent *k*-mer tiles, thus they cannot simply be compounded to get an accurate whole-read weight. We use several steps to determine which tiles represent significant bias and whether tiles are covariant or independent.

For each *k*-mer tile, we determine if it is significantly biased among all reads by comparing the average nucleotide variance to the variance observed in the baseline. Average nucleotide variance (*anv*) is calculated by computing the relative frequency of each nucleotide at each position in the *k*-mer tile (this is illustrated in Fig. 1), then computing the variance of each nucleotide across the *k*-mer tile and averaging them.

3 $$ anv_{t}=\frac {\sum\limits_{a\in {A,C,G,T}} {\frac {\sum\limits_{i\in [0,k]} {\left(f_{a}^{t+i}-\frac{\sum\limits_{j\in [0,k]}{f_{a}^{t+j}}}{k} \right)^{2}}} {k}}} {4}  $$

This produces, in visual terms, a measure of the “flatness” of nucleotide frequencies across the *k*-mer tile. If the average nucleotide variance is more than two standard deviations (95% confidence threshold) outside the variance in the baseline, we mark a tile as significantly biased. The identified biased tiles vary between assay types, although there is concordance between replicates and experiments using the same protocol (Fig. 2). As we noted previously, regions with significant bias are most often found surrounding the 5’ and 3’ ends of a read (Fig. 1).

To compute the covariance between a pair of biased tiles *A* and *B*, we enumerate the frequency of the *k*-mer at tile *A* and the *k*-mer at tile *B* for every read. We compute the coefficient of covariance between the tile *A* and tile *B* *k*-mer frequency vectors. The level of covariance is computed this way between every pair of biased tiles that we found in the previous step. We similarly compute an expected covariance measure between two equivalently spaced tiles in the baseline region.

We can combine these values into a matrix of covariance between all tiles. Additional file 1: Figure S1 illustrates a heat map of an example covariance matrix between non-overlapping tiles in DNase-seq data. This example indicates relatively high correlation between adjacent tiles surrounding the beginning of the read (tiles -1 and 0). These tiles straddle the DNase I binding site and are likely highly correlated because they reflect two halves of the preferred DNase I binding motif. We perform greedy nearest-neighbor clustering of biased tiles, joining tiles into covariance groups if their average pairwise covariance is significantly above the expected covariance computed from the baseline. We expect that the resulting clusters contain *k*-mer tiles that are dominated by bias from the same source, driving their correlation.

To compute the total weight for any sequence, we compute the adjustment value for each tile as the ratio between the frequency of the tile’s *k*-mer in the baseline and the observed frequency of the *k*-mer at the tile position, *t* is the index of the tile within the sequence, and *i* is the start position of the sequence in *S* (Eq. 4).

4 $$ w_{i}^{t}=\frac{b_{S_{[i+t, i+t+k]}}} {f_{S_{[i+t, i+t+k]}}^{t}}  $$

Per-tile weights are then aggregated according to the covariance groups. Tile weights within each group are averaged to best approximate the correction value from the source driving that group. Then whole-read weights are computed as the product of the weights from all groups, where *tileGroups* are the groups of tiles with significant pairwise covariance (Eq. 5):

5 $$ sequenceWeight_{i}=\prod\limits_{tiles\:\in\: tileGroups} \frac{\sum\limits_{t\:\in\:tiles} w_{i}^{t}} {|tiles|}  $$

The bias-corrected weight of a read is given in Eq. 6. To remove weight biases incurred due to the immediate genomic context of a read (ex. GC content), that is not consistent across the entire dataset, each read’s weight is normalized by the average weight of all read-length sequences within 10 bp of the observed read.

6 $$ readWeight_{r}=\frac{sequenceWeight_{A(r)}} {\left(\frac{\sum\limits_{j\in(-10,-1)\cup(1,10)} sequenceWeight_{A(r)+j}} {20} \right)}  $$

### Footprint and peak detection

We used protein interaction quantification (PIQ) [19] to predict transcription-factor binding sites. PIQ uses known binding motifs to explicitly identify the read pileup profile, “footprint”, associated with a transcription factor. Transcription factors CTCF, EP300, MAFK, RAD21, REST, and SP1 were analyzed. Motifs identified as a part of ENCODE [20] were input into FIMO (MEME suite [21]) with the following parameters: “–max-strand –max-stored-scores 1000000 –no-qvalue” to identify candidate binding sites in the hg19 reference genome. The output FIMO motif site predictions were then converted into BED format coordinates with the p-value and PWM score retained and blacklist filtered to remove sites in unalignable and repetitive regions. Sites were then filtered independently for each factor to remove those with a higher-confidence motif from a different transcription factor within 20 bp of the motif site. This filtered set of putative binding sites was used as input to PIQ, which output footprint confidence scores for each candidate site. To validate binding site predictions, positive sites are generated by overlapping all candidate sites with ENCODE ChIP-seq peak calls for the factor in question. These sites are further reduced by only allowing 1 motif site per peak. The site closest to the peak maximum is chosen. Negative sites must not overlap a peak call and have no ChIP-seq signal enrichment over baseline. PIQ scores and positive and negative groups are used to compute ROC curves and AUC values (Additional file 2: Table S1).

We identified open chromatin peaks in DNase-seq, ATAC-seq, FAIRE-seq, and ChIP-seq peaks using F-seq [4]. For each experimental dataset, we merged the BAM files for all independently bias-corrected replicates, then F-seq was run with the default parameters, outputting peaks in BED format. To run F-seq on our bias-corrected read data, we made simple modification to allow F-seq to parse and incorporate the included weight data into its model. Bias-corrected weights output by our method are included as a floating-point value using the optional tag “XW” in SAM/BAM format. Our fork of F-seq that includes this functionality to read the weight tag and incorporate floating-point weights is open source and can be found at http://github.com/txje/F-seq. We used this modified F-seq to predict peaks from our bias-corrected read data, using the default parameters.

## Results and discussion

To assess the impact of the weight corrections and to demonstrate generality across multiple assays, we calculated individual read weights for DNase-seq, FAIRE-seq, ATAC-seq, and ChIP-seq data from multiple human cell lines (GM12878, H1-hESC, and K562) generated within multiple laboratories as part of the ENCODE project [18] and in independent studies. We confirmed that our bias correction reduced the nucleotide variance in aggregate across all reads (Fig. 1 and Table 1). Adjusting *k*-mer frequencies to match the observed background frequency had the desired effect of driving the read-relative nucleotide frequencies toward the background level (Table 1). Encouragingly, this correction does not affect the global trends such as GC content, which, for instance, is known to be higher in transcriptionally active regions than in the genome at large. Preserving these assay-specific trends while eliminating bias at individual loci is an encouraging sign that we are not eliminating the signal with the bias.

**Table 1:** Average nucleotide variance before and after correction

	DNase-seq	FAIRE-seq	ChIP-seq	ATAC-seq
Raw data	0.022	0.006	0.005	0.023
Bias corrected	0.009	0.005	0.003	0.008

Variance before correction is especially high in DNase-seq and ATAC-seq as a result of DNase I and Tn5 binding preference, respectively

**Fig. 3:**
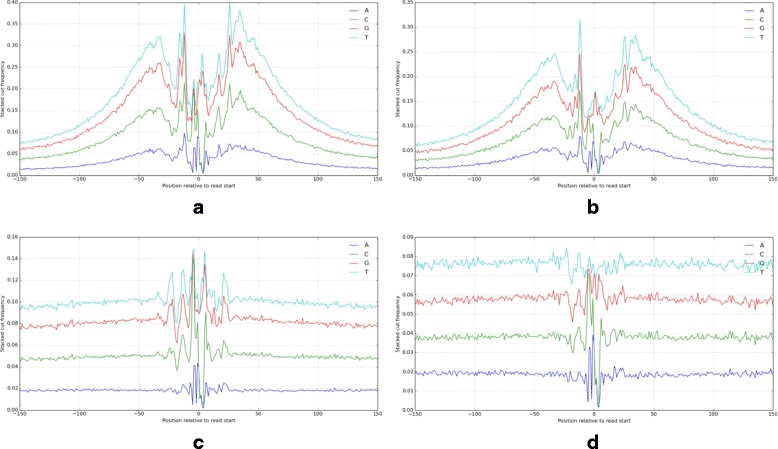
Aggregate stacked nucleotide pileups are shown across all reads within 250bp of known CTCF binding sites. DNase-seq data from GM12878 before and after bias correction are shown in (**a**) and (**b**), respectively. **c** and **d** show the cut profile on deproteinized “naked” K562 DNA before and after bias correction. In both cases, correction of nucleotide-specific bias removed spurious bias-driven spikes, smoothing the CTCF footprint (**b**) and restoring uniform coverage (**d**)

**Fig. 4:**
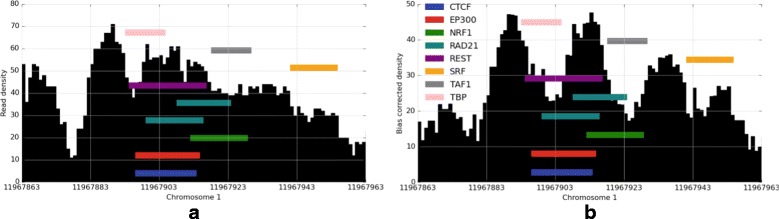
DNase-seq coverage across a hypersensitivity site on GM12878 chromosome 1. **a** shows the original raw cut density, **b** shows the cut density after bias correction, clarifying footprint of the bound transcription factors. Transcription factor binding motifs for 8 transcription factors are overlaid as *colored bars*

DNase-seq, FAIRE-seq, and ATAC-seq are commonly used to measure chromatin accessibility where transcription factors bind. These assays can be used to identify evidence of transcription factor binding [22, 23]. Binding sites often show a distinctly shaped depression, or footprint, in the distribution of read cut sites, evidence of an actively bound transcription factor impeding DNase I restriction or transposase insertion. Properties of different transcription factors influence the depth and shape of the footprint, particularly occupancy time [14]. However, footprints of high-occupancy factors such as CTCF provide an excellent case to study the effect of nucleotide-specific bias and our bias correction method on local features. We plotted the total DNase-seq read coverage surrounding predicted open CTCF binding sites before and after bias correction. Figure 3 illustrates the aggregate footprint profiles for GM12878 and deproteinized “naked” DNA from K562 samples. In the naked DNA, since all proteins influencing DNase I activity have been removed, we see only the effect of nucleotide-specific bias, driven largely by DNase I binding preference convolved with the CTCF binding motif. After bias correction, this signature is completely removed, restoring the uniform coverage we expect from deproteinized DNA. In GM12878, we see the peak with footprint depression in both original and bias corrected data. However, after bias correction, spurious peaks in the footprint profile are greatly reduced. The remaining spike is thought to be a reflection of the actual bound domain resulting from a gap in bound CTCF zinc fingers [24]. We show an example of a single DNase hypersensitive region with several TF binding sites in Fig. 4. This illustrates the clarification of the footprint shape at individual binding sites after bias correction and is particularly evident where the read density is high. An example of bias correction of ChIP-seq and DNase-seq reads in a superenhancer region is shown in Additional file 3: Figure S2.

We assessed the utility of bias correction to improve footprint identification by using protein interaction quantification (PIQ) [25] to predict transcription-factor binding sites in original and bias-adjusted GM12878 DNase-seq data. We found that PIQ better reflects the changes made by our bias correction because, unlike other footprinting methods, it explicitly models TF-specific footprint shape at a fine resolution. After correcting nucleotide-specific bias in these data, we were able to identify transcription factor binding sites (verified by ChIP-seq) with greater sensitivity and specificity than uncorrected data (Additional file 2: Table S1). Since PIQ explicitly models protein interactions with binding motifs, we saw different effects based on which motif occurred at a given site, with the greatest improvement at the most commonly bound motifs. Another confounding factor included the presence of proximal high-quality motifs for other transcription factors. Bias correction generally increases the total number of identifiable footprints, which, in many cases, causes false positives where motifs for multiple transcription factors occur close together. To avoid this, we considered only sites where the target factor has the most confident motif among common factors nearby. Of the factors we considered, only SP1 showed a decrease in specificity after bias correction. SP1 often acts as a recruiter for cofactors in promoter regions and is therefore very often coincident with other binding sites, which may cause an increase in false positives against the already very high sensitivity of PIQ for detecting SP1 binding sites (Additional file 2: Table S1).

To observe the effect of bias correction on open chromatin inference as a whole, we compared the covariance between DNase-seq, FAIRE-seq, ATAC-seq, and CTCF ChIP-seq from GM12878 cells under the same condition, but prepared and sequenced in different labs. Table 2 gives the coefficient of covariance between sequencing read depth across these experiments before and after bias correction. As expected, after correcting HTS- and assay-specific nucleotide bias, we observe consistent correlation among these experiments. Additionally, we called peaks using F-seq [4], which has been modified to use our read weights. Pairwise correlations were computed between the 50,000 highest scoring peaks from each data set (to reduce the effect of dramatically different read density across assays), also shown in Table 2. In five of six pairwise comparisons, the correlation between high-scoring peaks increases after bias correction using our method. The lone outlier, correlation between DNase-seq and FAIRE-seq peaks, may be confounded by the dramatically different read density and signal-to-noise ratios for these two assays.

**Table 2:** We computed the pairwise covariance between read densities in 250 bp windows and among weights of overlapping open chromatin peaks (using F-seq) before and after bias correction

	Read density		Peak weight	
	Raw	Corrected	Raw	Corrected
DNase vs. ChIP	0.2967	0.3112	0.3784	0.4138
DNase vs. FAIRE	0.3157	0.3105	0.6300	0.6029
DNase vs. ATAC	0.5268	0.5387	0.6620	0.6623
ChIP vs. FAIRE	0.1563	0.1589	0.2072	0.2214
ChIP vs. ATAC	0.2137	0.2182	0.2982	0.3138
FAIRE vs. ATAC	0.2700	0.2731	0.4637	0.4757

Correlations are shown between DNase-seq, FAIRE-seq, ATAC-seq, and ChIP-seq for a generic promoter, CTCF. Since these assays all target or are enriched in regions of open chromatin, we see convergence of these signals after correction

## Conclusion

We have shown that aggregate nucleotide-specific biases in high-throughput sequencing reads are greatly reduced by using our bias correction model. Reads are assigned weights to better represent their likelihood of occurrence in the absence of biases, regardless of the source of the bias. When our method is applied to epigenetic assays including DNase-seq, FAIRE-seq, ATAC-seq, and ChIP-seq, true open chromatin and transcriptionally active domains are more accurately identified.

Unlike previous methods focusing only on correction of DNase I restriction bias, our method is applicable to a wide range of HTS assays and conditions which may vary between lab, protocol specifics (including read length), cell type, and experimental condition. Existing methods to correct DNase-seq data apply read corrections based only on small motifs of 2-6 bp, often do not consider nucleotide biases outside the read boundaries, and/or require full sequencing of deproteinized “naked” DNA to identify DNase I and experimental biases [6, 13, 14]. Our proposed method corrects all bias within and surrounding reads, and without expensive additional sequencing.

While correlation between nucleotide-frequency-adjusted DNase-seq, ChIP-seq, FAIRE-seq, and ATAC-seq illustrates the generality of our method, there are several factors that may confound these correlations. Notably, many potentially bias-inducing steps during their respective HTS protocols are shared, particularly adapter ligation. We observe evidence of significant shared biases in the observed position-specific nucleotide frequencies, illustrated by the correlation between bias signatures under various experimental conditions (Fig. 2). Principal components analysis shows shared biases are correlated with assay type and lab/location, and may indicate other parameters, such as HTS technology-specific adapters. The same end-amplification adapters with similar binding preferences are often used for these assays. Thus, before correction, reads have similar biases, so the expected coincidence among reads between these protocols is overestimated. After nucleotide-specific adjustments using our method, bias-driven reads have been reduced, while their representation of true chromatin structure should have improved.

In most cases, popular peak-detection and variant detection methods can be trivially extended to use floating-point read weights. However, since we introduce new information about these HTS data with our bias-adjusted weights, these weights are not taken into account by default. In cases where this modification is not trivial, the weight data can be implicitly represented as variable integer copies of individual reads, thus increasing the amount of data that must be processed, but allowing this information to be used by existing analysis tools without modification.

## Additional files

Additional file 1 **Figure S1.** GM12878 DNase-seq 5-mer tile covariance matrix. The pairwise correlation is shown between bias values of 5-mer tiles in a 160bp window surrounding the 5’ end of aligned reads. The block structure between tiles 0 and 3 indicates correlation between adjacent k-mer frequencies within DNase-seq reads. (PDF 203 kb)

Additional file 2 **Table S1.** Area under curve (AUC) values for the ROC curves representing sensitivity and specificity of footprint detection for several transcription factors. AUC values at increasing false positive rates (FPR) are computed independently for each motif before and after correction. For all factors except SP1, bias correction improved our ability to accurately predict footprints using protein interaction quantification (PIQ), especially at low to moderate FPR. SP1 motifs often appear in promoters and coincide with binding sites for other factors, which may explain it’s high AUC and the increase in false positives caused by other detectable footprints after correction. (PDF 250 kb)

Additional file 3 **Figure S2.** ChIP-seq and DNase-seq coverage in a super enhancer region (Hnisz D, Abraham BJ, Lee TI, et al. Transcriptional super-enhancers connected to cell identity and disease. Cell. 2013;155(4):10.1016/j.cell.2013.09.053). This region is also in a DNase hypersensitivity region. We show both the ChIP-seq and DNase-seq signal before (A) and after (B) bias correction. In general, for regions with very high ChIP or DNase coverage like this and other “super enhancers”, bias correction doesn’t dramatically change the profile since peak and valley profiles are very robust. (PDF 327 kb)

## Abbreviations

ATAC: Assay for transposase-accessible chromatin
ChIP: Chromatin immunoprecipitation
ENCODE: Encyclopedia of DNA Elements
FAIRE: Formaldehyde-assisted isolation of regulatory elements
HTS: High-throughput sequencing
PCR: Polymerase chain reaction
PIQ: Protein interaction quantification
SDR: Short direct repeat
