# Supplementary material for: Correcting nucleotide-specific biases in high-throughput sequencing data
Source: BMC Bioinformatics. 2017 Aug 1;18:357. doi: 10.1186/s12859-017-1766-x (PMC5540620; doi:10.1186/s12859-017-1766-x)

Figure S1

GM12878 DNase-seq 5-mer tile covariance matrix. The pairwise correlation is shown between bias values of 5-mer tiles in a 160bp window surrounding the 5' end of aligned reads. The block structure between tiles 0 and 3 indicates correlation between adjacent k-mer frequencies within DNase-seq reads.

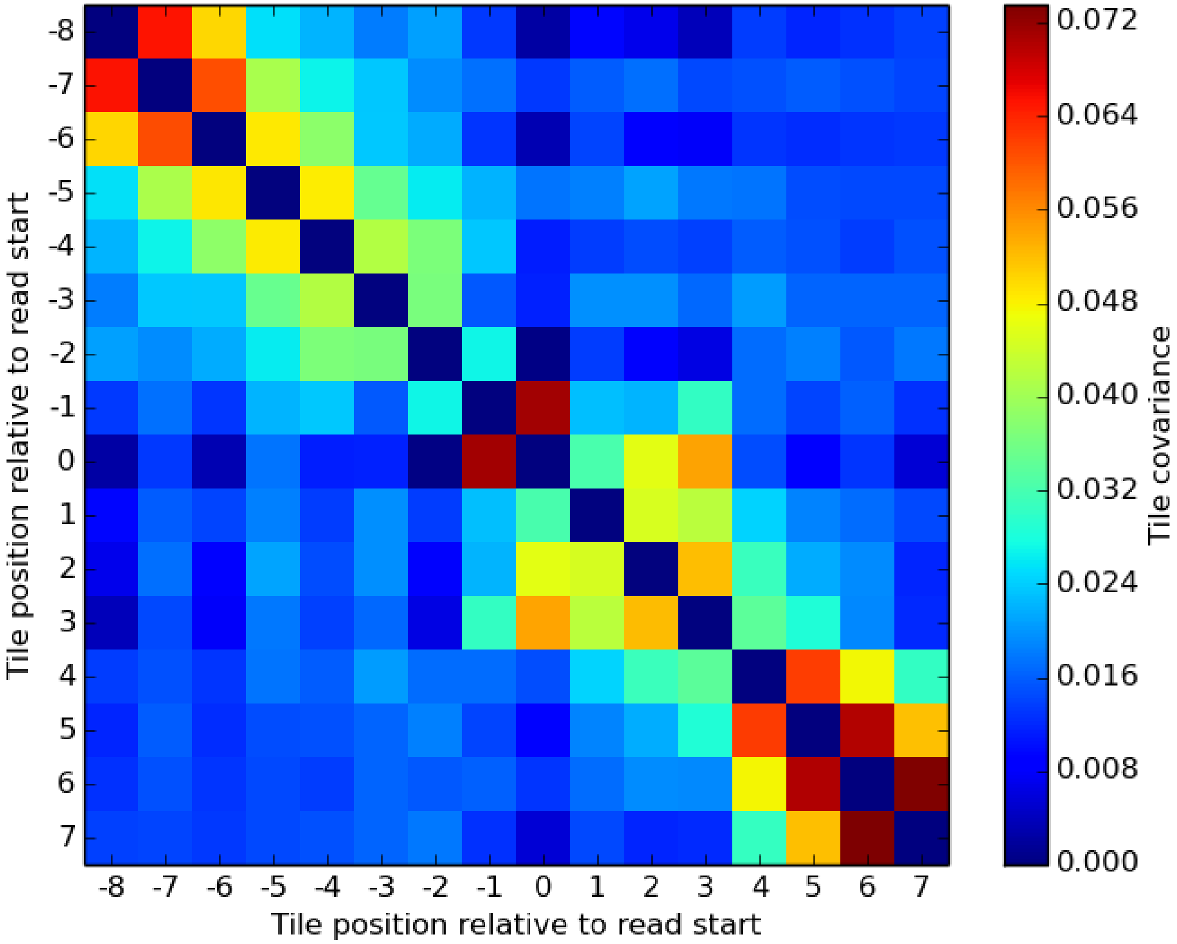

Supplement: Supplementary file 1 — Figure S1. GM12878 DNase-seq 5-mer tile covariance matrix. The pairwise correlation is shown between bias values of 5-mer tiles in a 160bp window surrounding the 5’ end of aligned reads. The block structure between tiles 0 and 3 indicates correlation between adjacent k-mer frequencies within DNase-seq reads. (PDF 203 kb) [file 12859_2017_1766_MOESM1_ESM.pdf]
