# Supplementary material for: Correcting nucleotide-specific biases in high-throughput sequencing data
Source: BMC Bioinformatics. 2017 Aug 1;18:357. doi: 10.1186/s12859-017-1766-x (PMC5540620; doi:10.1186/s12859-017-1766-x)
Supplement: Supplementary file 2 — Table S1. Area under curve (AUC) values for the ROC curves representing sensitivity and specificity of footprint detection for several transcription factors. AUC values at increasing false positive rates (FPR) are computed independently for each motif before and after correction. For all factors except SP1, bias correction improved our ability to accurately predict footprints using protein interaction quantification (PIQ), especially at low to moderate FPR. SP1 motifs often appear in promoters and coincide with binding sites for other factors, which may explain it’s high AUC and the increase in false positives caused by other detectable footprints after correction. (PDF 250 kb) [file 12859_2017_1766_MOESM2_ESM.pdf]

Table S1

Area under curve (AUC) values for the ROC curves representing sensitivity and specificity of footprint detection for several transcription factors. AUC values at increasing false positive rates (FPR) are computed independently for each motif before and after correction. For all factors except SP1, bias correction improved our ability to accurately predict footprints using protein interaction quantification (PIQ), especially at low to moderate FPR. SP1 motifs often appear in promoters and coincide with binding sites for other factors, which may explain it's high AUC and the increase in false positives caused by other detectable footprints after correction.

		AUC (FPR < 0.05)		AUC (FPR < 0.1)		AUC (FPR < 0.2)		AUC	
Transcription factor	Motif	Original	Bias corrected	Original	Bias corrected	Original	Bias corrected	Original	Bias corrected
CTCF	known1	0.0633	**0.102**	0.1423	**0.1961**	0.2657	**0.3449**	0.7416	**0.7975**
CTCF	known2	0.0257	**0.048**	0.068	**0.1015**	0.1558	**0.2191**	0.6897	**0.7521**
CTCF	disc1	0.0491	**0.0695**	0.1006	**0.1347**	0.2102	**0.272**	0.6947	**0.7564**
CTCF	disc2	0.131	**0.2088**	0.2395	**0.3194**	0.3673	**0.4521**	0.7803	**0.8317**
CTCF	disc3	0.0741	**0.1012**	0.1216	**0.1682**	0.2725	**0.3273**	0.7203	**0.7567**
CTCF	disc4	0.0924	**0.1215**	0.1573	**0.1945**	0.2617	**0.3008**	0.681	**0.7296**
CTCF	disc5	0.0555	**0.0857**	0.132	**0.1808**	0.2597	**0.3151**	0.7258	**0.7655**
CTCF	disc6	0.1066	**0.1088**	0.1465	**0.1612**	0.2332	**0.2522**	0.6474	**0.6646**
CTCF	disc7	0.0751	**0.0884**	0.1301	**0.1624**	0.2307	**0.2803**	0.6837	**0.7284**
CTCF	disc8	0.0773	**0.1035**	0.1556	**0.2005**	0.2915	**0.3453**	0.7095	**0.7526**
CTCF	disc9	0.0602	**0.0821**	0.1272	**0.1625**	0.2348	**0.2862**	0.703	**0.7525**
CTCF	disc10	0.0291	**0.0435**	0.0701	**0.1033**	0.1809	**0.2161**	0.6416	**0.6712**
RAD21	disc9	0.0228	**0.0252**	0.0751	**0.0802**	0.1552	**0.178**	0.6716	**0.675**
RAD21	disc3	0.0077	**0.0162**	0.0467	**0.0592**	0.1463	**0.157**	0.6505	**0.66**
RAD21	disc2	0.0354	**0.0419**	0.1028	**0.1155**	0.2079	**0.2224**	0.6689	**0.6865**
RAD21	disc4	0.0668	**0.0814**	0.1251	**0.144**	0.2274	**0.2489**	0.6895	**0.7002**
RAD21	disc5	0.0859	**0.1013**	0.1618	**0.1843**	0.276	**0.2979**	0.7054	**0.7213**
RAD21	disc6	0.0499	**0.0614**	0.1058	**0.1302**	0.2226	**0.2534**	0.694	**0.7136**
RAD21	disc8	0.0824	**0.1016**	0.1581	**0.1831**	0.2812	**0.3144**	0.7195	**0.7322**
RAD21	disc7	0.0631	**0.0774**	0.134	**0.1656**	0.2575	**0.2933**	0.7032	**0.7294**
RAD21	disc10	0.0903	**0.1103**	0.179	**0.1999**	0.319	**0.3403**	0.7474	**0.7628**
RAD21	disc1	0.0636	**0.0831**	0.1475	**0.1731**	0.2793	**0.3083**	0.738	**0.7554**
EP300	disc4	**0.1816**	0.147	0.3356	**0.3438**	0.5037	**0.526**	0.8328	**0.855**
EP300	disc9	**0.274**	0.2519	**0.4604**	0.373	**0.6133**	0.5296	**0.8893**	0.863
EP300	disc7	0.2488	**0.2925**	0.3608	**0.4035**	0.5152	**0.5388**	**0.8545**	0.8466
EP300	disc10	**0.2939**	0.2518	**0.4338**	0.3935	**0.5858**	0.5393	**0.8618**	0.827
EP300	disc3	0.2786	**0.2831**	0.3792	**0.3906**	0.4988	**0.5112**	**0.815**	0.8069

EP300	disc8	**0.2167**	0.1931	0.3683	**0.3836**	**0.5627**	0.5504	**0.8913**	0.853
EP300	disc1	**0.4215**	0.3993	0.4968	**0.4996**	0.5851	**0.6035**	**0.8643**	0.862
EP300	disc6	0.1781	**0.2664**	0.2545	**0.3294**	0.3704	**0.4362**	**0.7563**	0.7381
EP300	disc2	0.269	**0.2763**	0.3584	**0.373**	**0.4684**	0.4535	**0.8066**	0.7621
EP300	known1	0.2914	**0.3068**	0.419	**0.4349**	0.5484	**0.5777**	**0.8572**	0.8567
EP300	disc5	0.3336	**0.3881**	0.4689	**0.4968**	0.6019	**0.6104**	**0.8665**	0.8523
MAFK	disc1	0.1067	**0.1357**	0.1443	**0.1993**	0.2305	**0.3212**	0.6098	**0.6467**
MAFK	known8	**0.153**	0.1175	0.1919	**0.2126**	0.2407	**0.296**	**0.6115**	0.6059
MAFK	known5	0.0571	**0.0737**	0.0882	**0.1225**	0.1485	**0.1967**	**0.5657**	0.5571
MAFK	known7	0.0987	**0.1745**	0.1437	**0.2408**	0.2131	**0.3198**	0.6221	**0.6452**
MAFK	known9	0	**0.0208**	0	**0.1236**	0.0694	**0.1982**	0.4446	**0.5658**
MAFK	known6	0.0915	**0.1452**	0.1319	**0.1944**	0.241	**0.2865**	0.5747	**0.6075**
REST	known4	0.1141	**0.1394**	0.1625	**0.2091**	0.2297	**0.3171**	0.6062	**0.6678**
REST	disc7	0.5769	**0.619**	0.6691	**0.7362**	0.7606	**0.8119**	0.9151	**0.9219**
REST	disc9	0.4511	**0.4936**	0.5646	**0.6068**	0.6669	**0.7091**	0.8749	**0.8971**
REST	known1	0.2122	**0.2645**	0.2989	**0.3432**	0.4174	**0.4556**	0.7529	**0.7741**
REST	disc3	0.3642	**0.4028**	0.4404	**0.4832**	0.5089	**0.5592**	0.7713	**0.7936**
REST	disc2	0.2921	**0.3335**	0.3453	**0.3841**	0.4072	**0.4482**	0.6995	**0.7399**
REST	known2	0.6703	**0.7316**	0.7335	**0.7903**	0.8163	**0.8424**	0.9215	**0.9258**
REST	known3	0.2434	**0.2991**	0.3249	**0.3716**	0.4101	**0.4568**	0.7518	**0.7767**
REST	disc1	0.1498	**0.1534**	0.216	**0.2198**	0.3104	**0.3292**	0.7197	**0.7371**
REST	disc10	0.2927	**0.3397**	0.3818	**0.4544**	0.5041	**0.5817**	0.7957	**0.8256**
REST	disc6	0.4966	**0.5313**	0.6286	**0.6606**	0.7237	**0.752**	0.8716	**0.8743**
REST	disc8	0.4406	**0.494**	0.5259	**0.5895**	0.6138	**0.6736**	0.8283	**0.8486**
REST	disc5	**0.345**	0.3431	0.4323	**0.446**	0.5209	**0.5577**	0.7755	**0.7953**
REST	disc4	0.4915	**0.5247**	0.5721	**0.6037**	0.6441	**0.6897**	0.8551	**0.8825**
SP1	known5	**0.8253**	0.8119	**0.8571**	0.8488	**0.899**	0.8976	**0.964**	0.9639
SP1	disc3	**0.7061**	0.6787	**0.8064**	0.7874	**0.8774**	0.863	**0.9634**	0.9556
SP1	disc1	**0.3968**	0.3639	**0.6364**	0.6134	**0.7924**	0.7764	**0.9507**	0.9453
SP1	disc2	**0.6354**	0.6085	**0.7323**	0.701	**0.8119**	0.7846	**0.9439**	0.9296
SP1	known4	**0.5001**	0.4651	**0.6957**	0.664	**0.8208**	0.7985	**0.9518**	0.9448
SP1	known8	**0.6569**	0.6091	**0.7516**	0.7186	**0.8411**	0.8178	**0.953**	0.9462
SP1	known2	**0.6887**	0.6551	**0.7982**	0.7769	**0.8743**	0.8578	**0.9644**	0.9589
SP1	known7	**0.5722**	0.5555	**0.7253**	0.7057	**0.8296**	0.8156	**0.9533**	0.9498
SP1	known6	**0.5018**	0.4922	**0.7217**	0.6946	**0.8433**	0.8291	**0.9535**	0.951
SP1	known1	**0.7937**	0.7802	**0.8414**	0.8304	**0.8895**	0.876	**0.9636**	0.9568
