# Supplementary material for: Correcting nucleotide-specific biases in high-throughput sequencing data
Source: BMC Bioinformatics. 2017 Aug 1;18:357. doi: 10.1186/s12859-017-1766-x (PMC5540620; doi:10.1186/s12859-017-1766-x)
Supplement: Supplementary file 3 — Figure S2. ChIP-seq and DNase-seq coverage in a super enhancer region (Hnisz D, Abraham BJ, Lee TI, et al. Transcriptional super-enhancers connected to cell identity and disease. Cell. 2013;155(4):10.1016/j.cell.2013.09.053). This region is also in a DNase hypersensitivity region. We show both the ChIP-seq and DNase-seq signal before (A) and after (B) bias correction. In general, for regions with very high ChIP or DNase coverage like this and other “super enhancers”, bias correction doesn’t dramatically change the profile since peak and valley profiles are very robust. (PDF 327 kb) [file 12859_2017_1766_MOESM3_ESM.pdf]

Figure S2

ChIP-seq and DNase-seq coverage in a super enhancer region (Hnisz D, Abraham BJ, Lee TI, et al. Transcriptional super-enhancers connected to cell identity and disease. Cell. 2013;155(4):10.1016/j.cell.2013.09.053). This region is also in a DNase hypersensitivity region. We show both the ChIP-seq and DNase-seq signal before (A) and after (B) bias correction. In general, for regions with very high ChIP or DNase coverage like this and other “super enhancers”, bias correction doesn’t dramatically change the profile since peak and valley profiles are very robust.

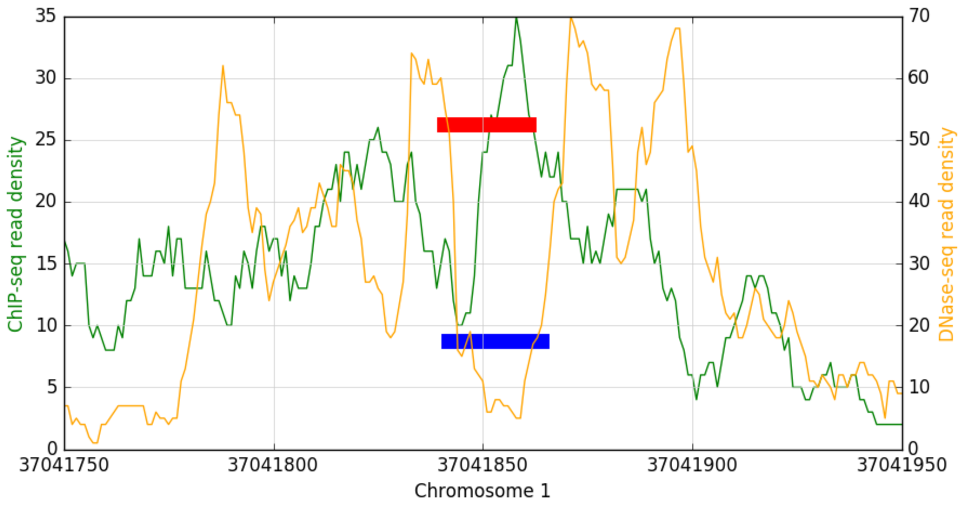

A

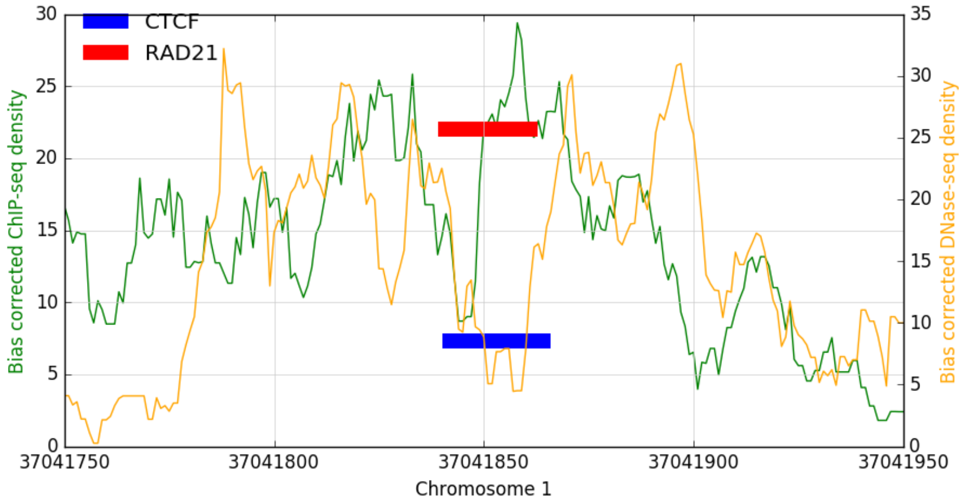

B
